# ADHS im Übergang: Wie gelingt Kontinuität in der Versorgung?

**DOI:** 10.1007/s00115-026-01956-5

**Published:** 2026-03-30

**Authors:** Sarah Hohmann, Alexandra Philipsen

**Affiliations:** 1 Klinik für Kinder- und Jugendpsychiatrie, -psychotherapie und -psychosomatik, Universitätsklinikum Hamburg-Eppendorf, Martinistr. 52, 20246 Hamburg, Deutschland; 2 Klinik und Poliklinik für Psychiatrie- und Psychotherapie, Universitätsklinikum Bonn, Bonn, Deutschland

**Keywords:** Transition, ADHS, Versorgung, Kooperation, Familie, Transition, ADHD, Healthcare service, Cooperation, Family

## Abstract

**Hintergrund:**

Der Übergang von der Kinder- und Jugendpsychiatrie (KJP) in die Erwachsenenpsychiatrie stellt für junge Menschen mit Aufmerksamkeitsdefizit‑/Hyperaktivitätsstörung (ADHS) eine vulnerable Phase dar. Unterschiedliche Versorgungsstrukturen, therapeutische Konzepte und Verantwortlichkeitsmodelle führen häufig zu Diskontinuitäten und Behandlungsabbrüchen.

**Ziel der Arbeit:**

Darstellung zentraler Herausforderungen der Transition bei ADHS sowie Ableitung praxisorientierter Empfehlungen zur Optimierung des Übergangs.

**Material und Methoden:**

Narrative Auswertung aktueller Leitlinien (Arbeitsgemeinschaft der Wissenschaftlichen Medizinischen Fachgesellschaften [AWMF], National Institute for Health and Clinical Exellence [NICE]), relevanter empirischer Literatur und qualitativer Studien zu Erfahrungen von Betroffenen, Familien und Behandelnden.

**Ergebnisse:**

Als wesentliche Barrieren zeigen sich begrenztes Wissen der Jugendlichen über ADHS und Behandlungsmöglichkeiten, ausgeprägte Schwierigkeiten im Selbstmanagement, fehlende interdisziplinäre Abstimmung, geringe Kenntnis der Angebote im Erwachsenenbereich und regionale Engpässe spezialisierter ADHS-Versorgung. Zusätzlich erschweren divergierende Behandlungskulturen der Fachdisziplinen sowie der Wegfall elterlicher Unterstützung nach Erreichen der Volljährigkeit den Übergang.

**Diskussion:**

ADHS ist eine persistierende neuroentwicklungsbedingte Störung und erfordert eine kontinuierliche, lebensspannenorientierte Versorgung. Eine gelingende Transition setzt strukturierte, standardisierte Prozesse, verlässliche Kommunikation zwischen den beteiligten Systemen, den angepasst fortgeführten Einbezug der Familie sowie spezialisierte Versorgungsangebote voraus. Zukünftige Modelle sollten entwicklungsorientierte und autonomiefördernde Ansätze von KJP und Erwachsenenpsychiatrie integrieren.

**Zusatzmaterial online:**

Die Online-Version dieses Beitrags (10.1007/s00115-026-01956-5) enthält eine Tabelle zu den aktuellen Leitlinienempfehlungen.

## Abstract (en)

**Background:**

The transition from child and adolescent to adult psychiatric care constitutes a high-risk period for young people with attention deficit hyperactivity disorders (ADHD). Differences in healthcare service structures, therapeutic concepts and responsibility allocation frequently result in discontinuity and termination of treatment.

**Aim:**

To summarize key challenges of ADHD transition and derive practice-oriented recommendations to improve continuity of care.

**Material and methods:**

Narrative review of current guidelines (AWMF, NICE), relevant empirical literature and qualitative studies addressing patient, family and clinician perspectives.

**Results:**

Major barriers include limited patient knowledge of ADHD, marked organizational difficulties, insufficient intersectoral coordination, lack of awareness of adult services and regional shortages of specialized ADHD care. Divergent treatment cultures and the abrupt reduction of parental involvement further complicate the process.

**Discussion:**

The ADHD is a persistent neurodevelopmental disorder requiring age-spanning, continuous care. Effective transition depends on structured procedures, reliable communication between services, developmentally adjusted family involvement and specialized treatment options. Future models should integrate developmental and autonomy-focused approaches from pediatric, juvenile and adult psychiatry.

## Hintergrund

Die mit der Versorgung psychisch erkrankter Kinder und Jugendlicher betrauten Helfersysteme (Kinder- und Jugendpsychiatrie und -psychotherapie, Jugendhilfe und Schule) operieren in Deutschland weiterhin weitgehend von den Systemen für Erwachsene (Psychiatrie, Psychotherapie und Psychosomatik, Arbeitsagentur und Eingliederungshilfe) getrennt. Dies bedingt nach dem Erreichen bestimmter Altersgrenzen (18 bzw. 21 Jahre) bei weiterer Behandlungsnotwendigkeit immer das Ende einer teils langjährigen Unterstützung und die Notwendigkeit der Überleitung in neue Behandlungs- und Unterstützungsformen. Gleichzeitig unterscheiden sich die Bereiche oft erheblich in ihrer therapeutischen Herangehensweise, einerseits mit starkem Entwicklungsbezug, Familienfokus, vielen pädagogischen Elementen und teils wenig Übergabe von Eigenverantwortung an die Betroffenen im KJ(Kinder/Jugendliche)-System, andererseits mit eher auf das Individuum fokussiertem, störungsspezifisch zugeschnittenem und viel Wert auf Selbstverantwortung und Freiwilligkeit legendem Vorgehen im Erwachsenensystem. Dass dieser Übergang eine Herausforderung darstellt, die für psychisch erkrankte junge Menschen schnell in einer Überforderung und damit einhergehend Behandlungsabbrüchen und teils mehrjähriger Nichtversorgung resultieren und zu einer Verschlechterung des psychosozialen Funktionsniveaus führen können, ist bereits langfristig bekannt und wurde innerhalb der beteiligten Fachgesellschaften bereits mehrfach adressiert [6, 14]. In Folge dessen sind in den letzten Jahren sektorenübergreifend zahlreiche interdisziplinäre transitionspsychiatrische Angebote entwickelt worden, um diesen Übergang besser zu gestalten und die sogenannte Transitionslücke schließen zu können. Die meisten dieser Konzepte fokussierten bisher auf störungsspezifische Angebote für Menschen mit früh beginnenden schweren psychischen Erkrankungen wie z. B. Schizophreniespektrumstörungen oder Persönlichkeitsstörungen [1], also klassischen Kernkompetenzbereichen der Psychiatrie. Wie aber gelingt der Übergang bei psychischen Erkrankungen, die lange als rein entwicklungsbezogene Phänomene des Kindes- und Jugendalters betrachtet wurden und bei denen der Ausbau von Kompetenzen und Versorgungsstrukturen im Erwachsenenbereich aktuell noch im Gange ist? Und unterscheiden sich die Bedarfe dieser Patient:innen und ihrer Familien und die daraus resultierenden Empfehlungen von den allgemein empfohlenen Vorgehensweisen für die Gestaltung des Überganges?

Die Aufmerksamkeitsdefizit‑/Hyperaktivitätsstörung (ADHS) ist mit einer Prävalenz von ca. 5 % [15] weltweit eine der häufigsten psychischen Erkrankungen bei Kindern und Jugendlichen. War das Konstrukt nach der International Statistical Classification of Diseases and Related Health Problems 10 (ICD 10) und dem Diagnostic and Statistical Manual of Mental Disorders IV (DSM IV) noch primär als Störung des Kindesalters angelegt, verbunden mit der Annahme einer weitgehenden Remission der Kernsymptomatik (bestehend aus Aufmerksamkeitsproblemen, motorischer Unruhe und Impulsivität) im Entwicklungsverlauf, ist inzwischen unbestritten, dass die Symptomatik über die Lebensspanne zwar bei den meisten Menschen insgesamt zurückgeht, ein wesentlicher Anteil (ca. 60 %) der Betroffenen aber auch im Erwachsenenalter noch die Diagnosekriterien erfüllt bzw. unter Einschränkungen des psychosozialen Funktionsniveaus leidet [10, 14, 21]. Die Prävalenz in der Bevölkerung liegt bei ca. 2,5 % [10]. Werden im Kindesalter noch deutlich mehr Jungen als Mädchen diagnostiziert, zeigt sich im Erwachsenenalter ein eher ausgeglichenes Geschlechterverhältnis. Diese Diskrepanz ist unter anderem durch Besonderheiten in der „weiblichen“ Ausprägung des ADHS-Phänotyps mit einer Betonung der unaufmerksamen, desorganisierten Komponente und weniger hypermotorischer oder auch disruptiver Symptomatik zu interpretieren, die seltener zu einer Zuweisung zur Diagnostik durch z. B. die Schule führt [23].

In der ICD 11 weist die Zuordnung der Diagnosekategorie ADHS zur neu geschaffenen Gruppe der neuromentalen Entwicklungsstörungen („neurodevelopmental disorders“) auf eine starke neurobiologische Komponente und einen frühen Beginn erster Vorläufersymptome hin, oft verbunden mit der Entwicklung einer oder mehrerer somatischer (u. a. erhöhtes Risiko für Übergewicht und Adipositas oder auch atopische Dermatitis und/oder Asthma bronchiale; [13]) und/oder psychiatrischer Komorbiditäten (z. B. mit einem erhöhten relativen Risiko [Odds Ratio, OR] von 4–5 für Angststörungen, depressive Störungen oder Suchterkrankungen sowie von > 9 für bipolare Störungen; [11]) über die Lebensspanne. Unbehandelt kann ADHS zu zahlreichen negativen psychosozialen Konsequenzen für die Betroffenen führen, z. B. in Bezug auf die Lebensqualität und den akademischen Erfolg, es bestehen zudem höhere Risiken für Unfälle, Suizidalität oder auch ungewollte Schwangerschaften [9].

Personen mit früh diagnostiziertem ADHS haben mit Erreichen des jungen Erwachsenenalters oft bereits mehrjährige Behandlungsphasen im kinder- und jugendpsychiatrischen und -psychotherapeutischen System hinter sich, bei vielen bestanden auch spezialisierte Hilfsangebote von Seiten der Jugendhilfe oder Unterstützung im schulischen Kontext bei gleichzeitig starker Involviertheit der betroffenen Familien. Hiermit einhergehend konnten oft entscheidende Entwicklungsaufgaben, z. B. im Sinne der Entwicklung von Kompetenzen zur Organisation eines eigenständigen Lebens nicht zeitgerecht gemeistert werden. Dass für diese Gruppe das Erreichen der Volljährigkeit, die damit einhergehende Beendigung bisheriger Hilfen, die gestiegenen Anforderungen in Bezug auf die Übernahme an Eigenverantwortung und die Überleitung in neue altersangemessene Angebote eine besonders vulnerable Phase darstellt, ist nachvollziehbar. Gleichzeitig gibt es auch eine wachsende Gruppe junger Menschen, vor allem auch junge Frauen, deren Symptomatik erst im Zusammenhang mit steigenden Anforderungen in Übergangsphasen (z. B. Schulabschluss, Beginn von Ausbildung oder Studium) deutlicher zutage tritt und die infolge dessen erst spät diagnostiziert werden [23]. Trotz anders gearteter Ausgangslage ist die Notwendigkeit einer möglichst kontinuierlichen Unterstützung in der vulnerablen Phase des Erwachsenwerdens auch bei dieser Klientel unbedingt gegeben.

**Abb. 1:**
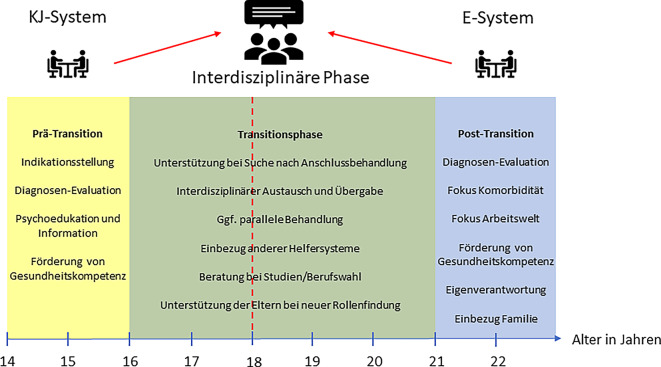
Überleitung in der Transitionsphase bei Aufmerksamkeitsdefizit‑/Hyperaktivitätsstörung unter Berücksichtigung von Leitlinienempfehlungen. *E* Erwachsene, *KJ* Kinder/Jugendliche

In der S3-Leitlinie „ADHS bei Kindern, Jugendlichen und Erwachsenen“ der Arbeitsgemeinschaft der Wissenschaftlichen Medizinischen Fachgesellschaften (AWWF; [4]) finden sich bereits in der Fassung von 2018 im Zusammenhang mit dem Transitionsprozess konkrete Empfehlungen zur Gestaltung des Überleitungsprozesses, die sich an der Leitlinie des National Institute for Health and Clinical Exellence (NICE; [4]) orientieren (siehe Tab. e1, Supplement). Vorgesehen sind hierbei unter anderem eine frühzeitige Identifizierung von Transitionsbedarfen, eine längerfristige Anbahnung und umfassende Psychoedukation, eine strukturierte Übergabe mit ggf. überlappender Behandlung sowie eine erneute diagnostische Evaluation nach Abschluss von Schule/Ausbildung (Abb. 1).

Gleichzeitig zeigen auch neuere Arbeiten, dass gerade bei jungen Menschen mit Entwicklungsstörungen wie ADHS die Transition oft nicht gut gelingt [22], also viele gar nicht erst im Erwachsenensystem ankommen oder die Phase des Überganges als schwierig und frustrierend erleben [5, 20]. So schätzte z. B. eine Studie aus Großbritannien, dass bei ca. 200 bis 500 17- bis 19-Jährigen mit ADHS auf 100.000 Einwohner ein Transitionsbedarf gegeben ist, tatsächlich aber nur bei ca. 38 bis 96 Betroffenen/100.000 in dieser Altersgruppe der Transitionsprozess erfolgreich beendet werden kann [7].

Dieser Diskrepanz zwischen übergeordneter Empfehlung und insuffizienter Umsetzung in der Versorgungspraxis liegen vermutlich zahlreiche Faktoren zugrunde, wie z. B. verwaltungstechnische und abrechnungsbedingte Barrieren, mangelndes Bewusstsein bezüglich des Bedarfes und der daraus resultierenden Notwendigkeit eines strukturierten gemeinsamen Vorgehens oder auch unzureichende interdisziplinäre Vernetzung und Information über das vorhandene Unterstützungsangebot im psychiatrischen Bereich.

## Patient:innenbezogene Faktoren, die Transition erschweren können

Grundvoraussetzung für eine gelingende Überleitung scheint eine umfassende Information und Psychoedukation der Betroffenen in Bezug auf ADHS zu sein, d. h. eine Aufklärung darüber, was Ursachen, Veränderung der Symptomatik im Entwicklungsverlauf, damit verbundene Risiken sowie Behandlungsempfehlungen (aber auch konkrete regionale Optionen) angeht. So ergeben qualitative Befragungen junger Menschen mit ADHS oft Hinweise auf große Unsicherheit und geringe Kenntnisse in Bezug auf das eigene Krankheitsbild und die damit verbundenen Unterstützungs- und Interventionsmöglichkeiten [18]. Auch Befragungen im Rahmen einer US-amerikanischen Studie zur Transition geben Hinweise auf ein patient:innenseitig im Vergleich mit den Eltern geringeres Bewusstsein für die Notwendigkeit der Fortführung der Therapie mit dem Eintritt ins Erwachsenenalter [12]. Dies kann, im Zusammenspiel mit einem einerseits stark ausgeprägten Wunsch nach Autonomie in dieser Lebensphase sowie gleichzeitig im Rahmen des ADHS evtl. ausgeprägt vorhandenen Schwierigkeiten in Bezug auf Organisations- und Selbstmanagementfähigkeiten [20], eine durch die Patient:innen selbst initiierte Suche nach einer geeigneten Anschlussbehandlung oder auch das Wahrnehmen von vereinbarten Anschlussterminen [7] deutlich erschweren.

Studien weisen darauf hin, dass Transition vor allem bei jungen Menschen mit schwer ausgeprägter Symptomatik und ggf. vorhandenen Komorbiditäten und daraus resultierend umfassender Unterstützung durch Familie und Helfersystem gelingt, dass aber bei eher mittelgradig bis mild ausgeprägter Symptomatik und Beeinträchtigung die betroffenen Jugendlichen im Übergangsalter sich mit der Aufgabe, den Übergang zu organisieren, eher allein gelassen und überfordert sehen [3].

## Auf die Behandler:innen bezogene und systembedingte Faktoren

Dass chronisch erkrankte Patient:innen und deren Familien nach teils jahrelangen Behandlungsverläufen im Bereich der Kindermedizin Unterstützung beim Übergang ins deutlich weniger umsorgende Erwachsenensystem benötigen und an die Übernahme der Verantwortung für das Management und die Behandlung ihrer Erkrankung erst langsam herangeführt und befähigt werden müssen, ist bereits langfristig bekannt. Es bestehen zahlreiche Empfehlungen hierzu, auch die meisten störungsbezogenen Leitlinien widmen dem Thema eigene Unterkapitel oder Empfehlungen (so auch z. B. die AWMF-Leitlinie ADHS [4]). In Deutschland wurde das Thema der Transition im psychiatrischen Bereich durch eine eigens eingerichtete Taskforce der Deutschen Gesellschaft für Psychiatrie und Psychotherapie, Psychosomatik und Nervenheilkunde (DGPPN) und der Deutschen Gesellschaft für Kinder- und Jugendpsychiatrie, Psychosomatik und Psychotherapie (DGKJP) im Jahr 2017 aufgegriffen und entsprechende Empfehlungen erarbeitet [6]. Diese beinhalten neben einem frühzeitigen Beginn der Planung und Aufklärung der Betroffenen und ihrer Familien einen gemeinsam gestalteten Übergangsprozess, der durch Abstimmung, Austausch und kollegiale Weitergabe von Informationen zwischen alten und neuen Behandler:innen, aber auch (wenn möglich) durch eine Phase der gleichzeitigen Behandlung geprägt ist.

Um den Übergang in einer solchen Form gestalten zu können, braucht es jedoch (neben Wissen auf Seiten der Behandelnden und auf die regionalen Besonderheiten angepassten Vorgaben) ein funktionierendes Netzwerk und eine bereits etablierte, gleichberechtigte und strukturierte Zusammenarbeit zwischen den beteiligten Partnerinstitutionen. Dies wird durch die weiterhin deutlich voneinander getrennten Behandlungsstrukturen im Kinder- und Erwachsenensystem, durch mögliche räumliche Distanz und unterschiedliche Trägerschaften von Kliniken sowie durch das auf beiden Seiten geringe Wissen um Angebote und Ansprechpartner im jeweils anderen Bereich erschwert. Auch die weggefallene Verpflichtung für angehende Kinder- und Jugendpsychiater:innen, während der Weiterbildung ein Jahr lang in einem Gegenfach wie z. B. der Psychiatrie zu arbeiten, trägt an vielen Standorten zu einer weiteren Verschlechterung des gegenseitigen Verständnisses bei. Gleichzeitig werden im psychiatrischen Bereich immer häufiger Angebote für junge Menschen als transitionspsychiatrisch eingerichtet, ohne den kinder- und jugendpsychiatrischen Blick auf Entwicklungsbezüge und familiäre Aspekte ausreichend einzubeziehen, was ebenfalls nicht hilfreich für die interdisziplinäre Zusammenarbeit sein kann. Hinzu kommt, dass in den letzten Jahren zwar zunehmend Angebote zur Diagnostik und Therapie für Erwachsene mit ADHS entstanden sind, die Versorgungslandschaft jedoch gleichzeitig weiterhin noch nicht ähnlich breit, vielseitig und verfügbar wie im Kinder- und Jugendbereich ausgebaut ist, was bei steigender Inanspruchnahme die Suche nach geeigneten spezialisierten Weiterbehandlungsplätzen zusätzlich erschwert. Auch entsprechen die psychiatrischen Behandlungsangebote, die oft stark auf medizinisch-pharmakotherapeutische Inhalte fokussieren, laut qualitativer Befragungen [17] nicht immer den Wünschen und Vorstellungen der jungen Betroffenen, die sich zu größeren Teilen eher spezialisierte psychosoziale bzw. psychotherapeutische Unterstützungsmöglichkeiten zum Start ins selbstbestimmte Erwachsenenleben erhoffen.

Neben diesen Aspekten spielt natürlich auch die auf allen Seiten hohe Termindichte, große Arbeitsbelastung und weiterhin größtenteils fehlende Finanzierung von Vernetzungs- bzw. Kooperationsbemühungen eine Rolle. So ergab z. B. eine Befragung unter KJP(Kinder- und Jugendpsychiatrie)-Behandelnden in Großbritannien [8] zwar Hinweise darauf, dass ein Großteil der Befragten die Empfehlungen der NICE-Leitlinie zur Gestaltung des Überganges zwischen KJP und Psychiatrie kennen und betroffene Jugendliche auch identifizieren und über die Notwendigkeit eines solchen Prozesses aufklären, weitere empfohlene Schritte wie eine detailliertere Abstimmung zwischen den Systemen oder auch die vorgesehene überlappende Behandlungsphase aufgrund von Kapazitätsgründen jedoch kaum zur Umsetzung kommen und deshalb auch in ihrer Sinnhaftigkeit angezweifelt werden. Auch eine mangelnde Übersetzung der übergeordneten Leitlinienempfehlungen in an die örtlichen Begebenheiten adaptierten Standard Operating Procedures (SOPs) bzw. Vorgaben kann dazu führen, dass die Thematik den an der Behandlung Beteiligten auf theoretischer Ebene durchaus geläufig ist, in der Praxis jedoch nicht zur Anwendung kommt [19].

## Der Einbezug der Familie

Der kontinuierliche, an den Entwicklungsstand des/der Betroffenen angepasste Einbezug von Familienmitgliedern in alle Entscheidungsprozesse und Schritte der Behandlung stellt ein Kernmerkmal einer kinder- und jugendpsychiatrischen Begleitung dar. Patient:innen mit ADHS werden oft bereits im Grundschulalter auf Initiative und Wunsch ihrer Eltern und Lehrkräfte erstmalig im KJP-System vorstellig und verbleiben nicht selten über Jahre und mehrere Entwicklungsstadien hinweg in dieser Konstellation. Hierbei nehmen die Eltern (ähnlich wie auch bei anderen chronischen Erkrankungen) oft eine stark unterstützende, organisierende und kontrollierende Funktion gegenüber ihrem Kind ein, um eventuell den durch die Kernsymptomatik verursachten Schwierigkeiten entgegenzuwirken bzw. diese abzupuffern. Mit Eintritt der Volljährigkeit und Wechsel in die Erwachsenenmedizin ändert sich die Vorgehensweise jedoch deutlich, primär wird nun der junge Mensch selbst in Verantwortung gesehen, der Einbezug der Angehörigen findet nicht mehr regelhaft statt. Dies kommt einerseits den Bedürfnissen und dem Autonomiebestreben der jungen Betroffenen entgegen, kann andererseits aber auch zu Überforderung auf Patient:innenseite und Frustration und Unverständnis auf Seiten der unterstützenden Eltern führen.

Ähnlich den Jugendlichen selbst, die im Entwicklungsverlauf verschiedene Entwicklungsaufgaben auf dem Weg zum Erwachsenwerden meistern müssen, durchlaufen auch Eltern im Zuge der Entwicklung ihrer Kinder einen äquivalenten Prozess, in dessen Rahmen eine langsame Ablösung und Übergabe von Verantwortung an die jungen Menschen erfolgen sollte. Im Zusammenhang mit ADHS ergab eine qualitative Studie, dass Eltern von ADHS-Patient:innen in früheren Stadien der Entwicklung und Behandlung oft eine „Manager-Rolle“ [12] einnehmen und ihre Kinder in allen Belangen ihres täglichen Lebens intensiv unterstützen und koordinieren. Im Entwicklungsverlauf bzw. im Übergang zum Erwachsenenalter ist es jedoch wichtig, ihre Rolle dahingehend zu modifizieren, eher supportiv im Hintergrund zu unterstützen, die Gesamtverantwortung für Behandlung wie auch allgemeine Koordination und Strukturierung aber sukzessive in die Hand der Betroffenen selbst zu übergeben („Roadie-Rolle“). Die Unterstützung der gesamten Familie im Ablösungsprozess und in der Annahme der jeweils veränderten Rolle scheint eine Aufgabe für alle am Transitionsprozess beteiligten Seiten zu sein und sollte in der Konzeption von Angeboten zur Unterstützung der Transition mitberücksichtigt werden.

## Diskussion und Implikationen auf theoretischer und praktischer Ebene

Die erläuterten Befunde unterstützen einmal mehr die Notwendigkeit der Betrachtung von ADHS im Sinne einer in der Kindheit beginnenden neuronalen Entwicklungsstörung, die im Gegensatz zu früher vorherrschenden Konzepten in der Regel nicht mit dem Eintritt ins Erwachsenenalter sistiert, sondern oft lebenslange Auswirkungen und Beeinträchtigungen und damit einhergehende Unterstützungsbedarfe mit sich bringt. Hieraus resultiert die Notwendigkeit der Entwicklung von lebensspannenübergreifenden Behandlungsansätzen mit besonderem Fokus auf einer Behandlungskontinuität über den Entwicklungsverlauf hinweg [2]. Gleichzeitig lässt sich der Prozess der Transition nicht auf wenige einzelne Empfehlungen herunterbrechen, die eine einfache und allgemeingültige Umsetzung ermöglichen, sondern stellt immer einen komplexen und vielschichtigen, individuell am Entwicklungsstand sowie den psychosozialen Umständen und Bedarfen des jeweiligen Betroffenen orientierten Vorgang dar, der einer guten ausführlichen Vorbereitung und Abstimmung zwischen den beteiligten Systemen bedarf [17, 24]. Hierbei spielt auch die Unterstützung des Familiensystems und deren Veränderung im Rahmen des Entwicklungsprozesses von einer organisierenden Kraft hin zu einer eher empowernden/bestärkenden Position eine besondere Rolle [12]. Auch geschlechtsspezifische Unterschiede bezüglich bestehender Symptomatik, Komorbiditäten, hieraus resultierenden Beeinträchtigungen und daraus abgeleiteten differenzierten Behandlungsempfehlungen sollten in die Konzeption transitionspsychiatrischer Angebote einfließen.

Zukünftige Versorgungsmodelle für Jugendliche und junge Erwachsene mit ADHS sollten zudem die unterschiedlichen Behandlungsphilosophien und Herangehensweisen der Fachdisziplinen KJP (eher entwicklungsbezogen und familienzentriert) und Psychiatrie (eher auf die Autonomie des Individuums fokussierend) berücksichtigen und miteinander zu integrieren versuchen, um eine wirkliche und effektive Brückenfunktion einnehmen zu können [17]. Vor allem die Weitergabe von Informationen und eine konstant gute Kommunikation zwischen den einzelnen Stakeholdern (Patient, Familie, unterschiedliche Behandlungssysteme) scheinen als Mediatoren für eine gelingende Transition zu wirken, im Einklang mit Theorien, die vor allem Faktoren wie Hintergrundwissen, Selbstwirksamkeit und Empowerment (im Sinne einer Hilfe zur Selbsthilfe?) als essenzielle Voraussetzungen für ein gelungenes Management chronischer Erkrankungen einordnen [16]. Deutlich wird aus der Literatur zudem, dass die oft nicht an den Wünschen und Bedarfen der jungen Betroffenen ausgerichtete und regional teils nur sehr limitiert verfügbare spezialisierte Versorgung für erwachsene Patient:innen mit ADHS einen weiteren gewichtigen Faktor im Zusammenhang mit nicht gelingenden Transitionsprozessen ausmacht. Dies impliziert auf Ebene des Versorgungssystems evtl. notwendige Maßnahmen im Sinne z. B. einer Ressourcenallokation hin in spezialisiertere Bereiche oder einer Verbesserung der fachlichen Expertise klinischer Mitarbeitender und Teams durch eine stärkere und verpflichtende Verankerung der Thematik in Aus‑, Fort- und Weiterbildung.

## Fazit für die Praxis

- Um die Versorgung junger Menschen mit Aufmerksamkeitsdefizit‑/Hyperaktivitätsstörung (ADHS) im Übergangsalter nachhaltig zu verbessern, bedarf es klar strukturierter, lokal adaptierbarer Standard Operating Procedures mit definierten Zuständigkeiten und Zeitabläufen.
- Die fachliche Expertise zu ADHS im Erwachsenenalter sollte durch gezielte Fortbildungen in der Psychiatrie ausgebaut werden und gleichzeitig sollten transitionsrelevante Inhalte stärker in der kinder- und jugendpsychiatrischen Weiterbildung verankert sowie durch aktive Netzwerkarbeit ergänzt werden.
- Ein den Entwicklungsaufgaben angemessener Einbezug der Familie ist notwendig, der fortbestehende Unterstützungsbedarfe berücksichtigt und gleichzeitig die veränderten Rollen im Übergang reflektiert.
- Informationsvermittlung und Kommunikation müssen durch niedrigschwellige, partizipativ erarbeitete Materialien und verlässliche Kommunikationswege verbessert werden.
- Die Versorgung erfordert einen Ausbau spezialisierter, multimodaler Angebote für junge Erwachsene sowie flexiblere, am individuellen Entwicklungsstand orientierte Altersgrenzen im Übergangsbereich. Hierbei sollten besondere Bedarfe z. B. von weiblichen Betroffenen Berücksichtigung finden.

## Supplementary Information

**Tabelle e1** Aktuelle Leitlinienempfehlungen aus der AWMF S3-Leitlinie, modifiziert und konsentiert im Rahmen des Leitlinienupdates 2025, Expertenkonsens
