# Supplementary material for: ADHS in transition: how can continuity in treatment succeed?
Source: Nervenarzt. 2026 Mar 30;97(3):239–45. [Article in German] doi: 10.1007/s00115-026-01956-5 (PMC13171936; doi:10.1007/s00115-026-01956-5)
Supplement: Supplementary file 1 — Tabelle e1 Aktuelle Leitlinienempfehlungen aus der AWMF S3-Leitlinie, modifiziert und konsentiert im Rahmen des Leitlinienupdates 2025, Expertenkonsens [file 115_2026_1956_MOESM1_ESM.pdf]

Tabelle e1 Aktuelle Leitlinienempfehlungen aus der AWMF S3 Leitlinie, modifiziert und konsentiert im Rahmen des Leitlinienupdates 2025, Expertenkonsens (Supplement)

***Leitfrage 1.6.1**:*<br><br>*Bei welchen Patienten und durch wen soll eine Transition zur Weiterbehandlung im Erwachsenenalter erfolgen?*
**Empfehlung**:<br><br>Bei sich in kontinuierlicher Behandlung befindenden Patient*innen mit ADHS sollte bei Erreichen des Transitionsalters (16-21 Jahre) eine erneute Evaluation bezüglich Symptomatik, koexistierender Störungen und Beeinträchtigungen erfolgen, um die Notwendigkeit einer weiteren längerfristigen Behandlung abschätzen und entsprechende Empfehlungen abgeben zu können. Eine Überweisung an qualifizierte weiterbehandelnde Ärzt*innen/Psychotherapeut*innen sollte bei entsprechender Indikation und Wunsch der Patient*innen angeboten werden.
Leitfrage 1.6.2:<br><br>*Wie sollte die Transition erfolgen?*
**Empfehlung:**<br><br>Wenn eine Fortführung der Behandlung notwendig ist, sollten die weiterbehandelnden Ärzt*innen / Psychotherapeut*innen durch die Vorbehandler*innen umfassend über Vorgeschichte, Behandlungsverlauf und derzeitiges Krankheitsbild informiert werden, um einen möglichst fließenden Übergang zu ermöglichen. Auch während des Transitionsprozesses sollten Absprachen zwischen den vor- und weiterbehandelnden Ärzt*innen /Psychotherapeut*innen ermöglicht oder eingeplant werden. Während der Transition sollten mit den Betroffenen Gespräche zur Vermittlung von umfassenden Informationen über die Versorgung im Erwachsenenbereich und zur Erfassung von Ängsten/Wünschen/Unsicherheiten und individuellen Unterstützungsbedarfen im Sinne eines partizipativen Vorgehens geführt werden.
Leitfrage 1.6.2:<br><br>***Welche primären Maßnahmen sollten bei den weiterbehandelnden Ärzten/Psychotherapeuten erfolgen?***
**Empfehlung:**<br><br>Bei den weiterbehandelnden Ärzt*innen / Psychotherapeut*innen sollte entweder direkt zum Zeitpunkt der Überleitung oder spätestens mit Abschluss der Schule/Ausbildung eine erneute umfassende Untersuchung der Patient*in mit ADHS erfolgen, die die Erfassung des ausbildungsbezogenen, beruflichen und sozialen Funktionsniveaus einschließt. Zusätzlich sollten bei entsprechenden Hinweisen koexistierende Störungen abgeklärt werden, vor allem Persönlichkeitsstörungen, Substanzmissbrauch und Störungen der Emotionsregulation.
